# Detection of European Strain of *Echinococcus multilocularis* in North America

**DOI:** 10.3201/eid1806.111420

**Published:** 2012-06

**Authors:** Emily J. Jenkins, Andrew S. Peregrine, Janet E. Hill, Christopher Somers, Karen Gesy, Brian Barnes, Bruno Gottstein, Lydden Polley

**Affiliations:** University of Saskatchewan, Saskatoon, Saskatchewan, Canada (E. Jenkins, J.E. Hill, K. Gesy, L. Polley);; University of Guelph, Guelph, Ontario, Canada (A. Peregrine);; University of Regina, Regina, Saskatchewan, Canada (C. Somers);; Westview Veterinary Hospital, Powell River, British Columbia, Canada (B. Barnes);; Universitat Bern, Bern, Switzerland (B. Gottstein)

**Keywords:** Echinococcus, Echinococcus multilocularis, alveolar hydatid cyst, zoonoses, European strain, North America, Canada, parasites, cestodes, dogs, canine, canid

**To the Editor:** In 2009, an alveolar hydatid cyst, the intermediate stage of the cestode *Echinococcus multilocularis*, was detected in the liver of a dog from Quesnel, British Columbia (BC), Canada (*1*), 600 km west of the nearest known record of this parasite in central North America (Figure). Alveolar hydatid cysts normally occur in rodent intermediate hosts. However, humans can serve as aberrant intermediate hosts; cysts generally originate in the liver and, in about one third of cases, metastasize throughout the body (*2*). Detection of the larval stage of this pathogen in an unusual host in a new geographic region required application of multiple molecular epidemiologic techniques to determine if this was range expansion of a native strain or introduction of a new strain of veterinary and public health concern.

**Figure:**
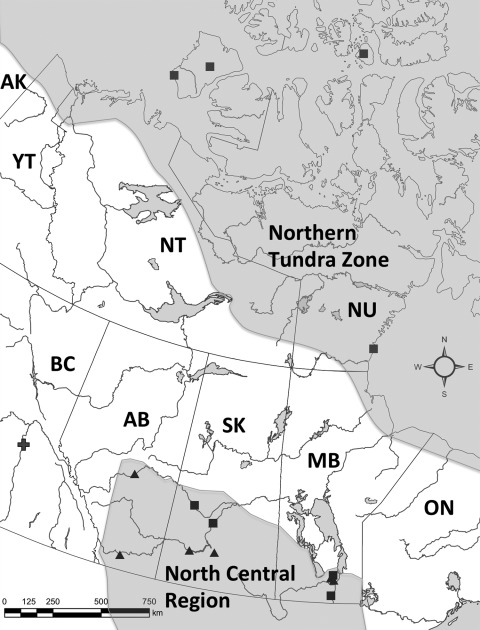
Location where European-type strain of *Echinococcus multilocularis* (plus sign) was detected in this study in British Columbia (BC) and previous reports of *E. multilocularis* parasites in 8 definitive (squares) and 6 intermediate (triangles) hosts in Canada. Gray shading indicates currently accepted distribution of *E. multilocularis* in North America. The North Central Region includes southern portions of the 3 Canadian prairie provinces (Alberta [AB], Saskatchewan [SK], and Manitoba [MB]) and 12 contiguous north-central US states (not shown). The western portion of the Northern Tundra Zone is based roughly on the established distribution of Arctic fox in Alaska (AK), the Yukon Territory (YT), Northwest Territories (NT), Nunavut (NU), northern MB, and northern Ontario (ON).

Alveolar hydatid cyst material was surgically excised from the dog, frozen, and shipped to the Western College of Veterinary Medicine in Saskatoon, Saskatchewan. DNA was extracted by using the QIAGEN DNeasy Blood and Tissue Kit (QIAGEN, Inc., Valencia, CA, USA). PCR was performed by using primers for 4 mitochondrial loci: NADH dehydrogenase subunit 1 (*nad1*) and 2 (*nad2*), cytochrome b (*cob*), and cytochrome c oxidase subunit 1 (*cox1*) (*3**,**4*). Sequence of a 488-bp region of the *nad1* gene (GenBank accession no. JF751034) was 99%–100% identical to *E. multilocularis* sequences from Asia (AY389984) and Europe (AB668376). Sequence data for 91 of 112 positions at the *cox1* (partial), *cob1*, and *nad2* genes (GenBank accession nos. JF751033, JF751035, and JF751036) grouped with haplotypes from Europe (*4*); 2 nucleotide differences from the E4 haplotype in foxes in France and Belgium were found, and 1 additional nucleotide difference (position 663 in *cob1*) did not correspond to any of the haplotypes defined previously.

Two independent subsamples of cyst material were fixed in 70% ethanol and shipped to the University of Regina, Regina, Saskatchewan, for PCR using primers targeting microsatellite loci EmsB and NAK 1 (*5**,**6*). PCR products were sized with single-basepair resolution by using capillary electrophoresis on a DNA sequencer (Genome Lab GeXP; Beckman-Coulter, Fullerton, CA, USA). Peaks that had <15% of the amplitude of the highest peak were excluded from analysis. EmsB electrophoregrams from the 2 samples of cyst material were identical and displayed 10 peaks spanning 220–238 bp. Visually, the EmsB profile from the BC dog sample most closely matched European profile H from foxes in west-central Europe (*5*). The BC dog sample was homozygous for the 198-bp allele at NAK 1, a genotype found in a variety of locations in Europe and Japan but not in North America, where the dominant genotype appears to be homozygous 192 (*5*).

Mitochondrial and microsatellite characterization showed that the genotype of *E. multilocularis* found in the BC dog was most similar to those described from west-central Europe. If the BC report represented a westward range expansion of a native North American strain of *E. multilocularis*, it would most likely be the N2 strain, established in the North Central Region, which includes the 3 Canadian prairie provinces and 12 contiguous north central American states (*4*) (Figure). This case demonstrates the utility of molecular epidemiology for detecting incursion of foreign pathogens and tracing their origins, as well as the feasibility of using animal sentinels to detect the introduction of a disease of public health concern into a new area.

Because the dog had never left BC and cestode eggs were not detected from a single fecal sample examined on microscopy (*1*), infection most likely resulted from consumption of infective eggs in the feces of a carnivore-definitive host. This host could have been a translocated domestic dog, thought to be the mechanism of recent introduction of *E. multilocularis* parasites into Sweden (*7*). It is also possible that a European strain of the parasite was introduced into North America in the last century, when red fox from France and Scandinavia were introduced (*8*).

The possible establishment of a European strain in North American wildlife, with spillover into domestic dogs, may have implications for public health and require increased vigilance by medical and veterinary personnel in the newly endemic region. Compared with native North American strains, European strains of *E. multilocularis* appear to have greater potential to cause alveolar hydatid disease (AHD) in humans. These strains are emerging worldwide (increasing in both prevalence and distribution) as a result of changes in landscape, climate, and wildlife–human interfaces (*2**,**9**,**10*). In Europe, human AHD can be fatal (definite or probable cause of death in 23.5% of 119 recent cases) and has low cure rates (5% of 408 recent cases) (*2*). As of 2000, in Europe and Asia, the estimated cost per case of AHD was US $100,000–$300,000 (*9*). Therefore, better understanding of the distribution, genetic diversity, and pathogenicity of strains of *E. multilocularis* is needed to assess risks and mitigate costs for public and veterinary health, as well as to provide evidence for the regulation and screening of imported domestic animals and translocated wildlife.
